# Dynamic Strain Measured by Mach-Zehnder Interferometric Optical Fiber Sensors

**DOI:** 10.3390/s120303314

**Published:** 2012-03-08

**Authors:** Shiuh-Chuan Her, Chih-Min Yang

**Affiliations:** Department of Mechanical Engineering, Yuan Ze University, 135 Yuan-Tung Road, Chung-Li 320, Taiwan; E-Mail: s945061@mail.yzu.edu.tw

**Keywords:** optical fiber sensor, Mach-Zehnder interferometer, dynamic strain, 3 × 3 coupler

## Abstract

Optical fibers possess many advantages such as small size, light weight and immunity to electro-magnetic interference that meet the sensing requirements to a large extent. In this investigation, a Mach-Zehnder interferometric optical fiber sensor is used to measure the dynamic strain of a vibrating cantilever beam. A 3 × 3 coupler is employed to demodulate the phase shift of the Mach-Zehnder interferometer. The dynamic strain of a cantilever beam subjected to base excitation is determined by the optical fiber sensor. The experimental results are validated with the strain gauge.

## 1. Introduction

Optical fiber sensors have attracted considerable attention in recent years as powerful measurement devices. They have been used in a variety of engineering applications such as residual strain measurement in composites [1], thin film stress measurement [2], monitoring mixed mode cracks [3], and gas detection [4]. Due to their small size and light weight, optical fiber sensors are appropriate for embedding or surface bonding to the structures. Optical fiber sensors can be classified according to the light parameters that are modulated. They are three types of sensors: the intensity, the phase and the wavelength modulated [5].

Vibrations have a significant effect on the fatigue life of structures and may even have disastrous consequences. To understand the vibrating behavior of structures, instrumentation for accurate vibration measurement is essential. A number of sensors are available for the measurements of a vibrating structure including strain gauge, piezoelectric transducer, laser vibrometer, accelerometer and optical fiber sensor. Among these measurement devices, optical fiber sensors have received much attention for structural health monitoring applications. They are unique in a number of aspects including small physical size, ease of embedment in structures, immunity to electromagnetic interference and excellent multiplexing capabilities [6], which make them ideal for vibration measurement. The fiber Bragg grating (FBG) and extrinsic Fabry-Perot interferometer (EFPI) techniques have been successfully demonstrated for the measurement of structural dynamic responses. Jin *et al.* [7] used fibre-optic grating sensors for flow induced vibration measurement. Betz *et al.* [8] developed a damage localization and detection system based on fiber Bragg grating Rosettes and lamb waves. Kirkby *et al.* [9] demonstrated the localization of impact in carbon fiber reinforced composites using a sparse array of FBG sensors. Frieden *et al.* [10,11] presented a method for the localization of an impact and identification of an eventual damage using dynamic strain signals from fiber Bragg grating (FBG) sensors. EFPI sensors have been used for strain measurement [12,13] and acoustic emission [5,14,15]. The EFPI sensor is more sensitive than the FBG, but the demodulation required high cost methodologies.

In this work, Mach-Zehnder optical fiber interferometric sensor is employed to measure the dynamic strain of a vibrating cantilever beam. The method developed by Brown *et al.* [16,17] for demodulation of the phase shift is adopted. The demodulation scheme utilizes a 3 × 3 coupler to reconstruct the signal of interest. The demodulation scheme has the advantage of passive detection and low cost as it requires no phase or frequency modulation in reference arm [18]. The experimental test results are validated with the strain gauge.

## 2. Mach-Zehnder Interferometer

**Figure 1.**
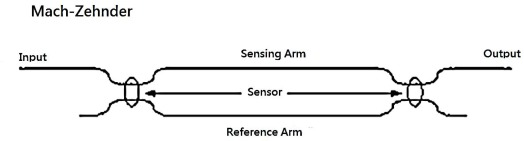
Mach-Zehnder interferometer.

The schematic diagram of a Mach-Zehnder interferometer is shown in Figure 1. It consists of two 2 × 2 couplers at the input and output. The excitation is applied to the sensing fiber, resulting optical path difference between the reference and sensing fibers. The light intensity of the output of the Mach-Zehnder interferometer can be expressed as [19]:

(1) $$\begin{array}{c} I=2A^{2}(1+\text{cos}\Delta \phi )\\ \Delta \phi =\frac{2\pi n_{0}}{\lambda }\left\{1-\frac{n_{0}}{2}\left[(1-v)P_{12}-v_{f}P_{11}\right]\right\}\int_{L_{f}}\;\varepsilon_{f}\;\mathrm{dx} \end{array}$$

where Δ*ϕ* is the optical phase shift; *n*_0_ is the refractive index of the optical fiber; *λ* is the optical wavelength, *v_f_* is the Poisson’s ratio; *P*_11_ and *P*_12_ are the Pockel’s constants; *L_f_* and *ε_f_* are the length and strain of the optical fiber, respectively. Since the terms in front of the integral sign of Equation (1) are constants for any given optical fiber system, the total optical phase shift Δ*ϕ* is proportional to the integral of the optical fiber strain. By measuring the total optical phase shift, the integral of the optical fiber strain can be easily obtained as follows:

(2) $$\int_{\Gamma_{S}}\varepsilon_{f}\;\mathrm{dx}=\frac{\Delta \phi }{\frac{2\pi n_{0}}{\lambda }\left\{1-\frac{1}{2}n_{0}^{2}\left[(1-v_{f})P_{12}-v_{f}P_{11}\right]\right\}}$$

The integral of the strain in Equation (2) denotes the change of the length of the sensing fiber which is surface bonded onto the host structure. The average strain of the optical fiber for optical phase shift Δ*ϕ* is:

(3) $$\varepsilon_{\mathrm{avg}}=\frac{\int_{\Gamma_{S}}\;\varepsilon_{f}\mathrm{dx}}{L_{f}}=\frac{\lambda \Delta \phi }{2L_{f}\pi n_{0}\left\{1-\frac{1}{2}n_{0}^{2}\left[(1-v_{f})P_{12}-v_{f}P_{11}\right]\right\}}$$

Thus, once the phase shift Δ*ϕ* of the Mach-Zehnder interferometer is demodulated, the strain of the host structure can be determined by utilizing Equation (3).

## 3. Demodulation of Phase Shift

**Figure 2.**
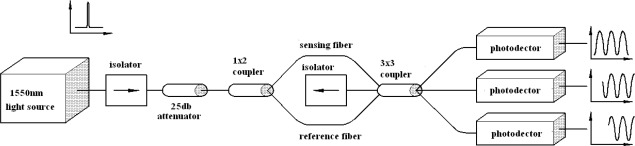
Schematic diagram of the Mach-Zehnder interferometric optical fiber sensor.

To demodulate phase shift Δ*ϕ* of the Mach-Zehnder interferometer, a 3 × 3 coupler is employed. Figure 2 shows the schematic diagram of the demodulation scheme. It consists of a 1 × 2 coupler at the input and a 3 × 3 coupler at the output. The two outputs of the 1 × 2 coupler comprise the reference fiber and sensing fiber of the Mach-Zehnder interferometer. The sensing fiber is surface bonded onto the host structure. Mechanical or thermal loadings applied to the host structure, leads to an optical path difference between the two fibers. The difference in the optical path induces a relative phase shift in the Mach-Zehnder interferometer. The two optical signals are guided into two of the three inputs of a 3 × 3 coupler, where they interfere with one another. The methodology developed by Brown *et al.* [16,17] for demodulation of the phase shift is adopted and briefly described as follows.

The three outputs of the 3 × 3 coupler are nominally 120° out of phase with either of its neighbors and can be expressed as:

(4a) $$x_{1}=C+B\text{cos}[\Delta \phi (t)]$$

(4b) $$x_{2}=C+B\text{cos}[\Delta \phi (t)-120{}^{\circ}]$$

(4c) $$x_{3}=C+B\text{cos}[\Delta \phi (t)+120{}^{\circ}]$$

where subscripts 1, 2 and 3 denote the three outputs of the 3 × 3 coupler, respectively; Δ*ϕ*(*t*) is the phase shift between the sensing and reference fibers of the Mach-Zehnder interferometer; C is the central value around which the output will vary with amplitude B.

The DC offset “C” of the output can be obtained by adding the three inputs as follows:

(5a) $$x_{1}+x_{2}+x_{3}=3C+B\{\text{cos}[\Delta \phi (t)]+\text{cos}[\Delta \phi (t)+120{}^{\circ}]+\text{cos}[\Delta \phi (t)-120{}^{\circ}]\}=3C$$

(5b) $$C=\frac{1}{3}(x_{1}+x_{2}+x_{3})$$

Three new parameters, *y*_1_, *y*_2_ and *y*_3_ are introduced as follows:

(6a) $$y_{1}=x_{1}-C=B\text{cos}[\Delta \phi (t)]$$

(6b) $$y_{2}=x_{2}-C=B\text{cos}[\Delta \phi (t)-120{}^{\circ}]$$

(6c) $$y_{3}=x_{3}-C=B\text{cos}[\Delta \phi (t)+120{}^{\circ}]$$

The next step in the processing is to take the difference between each of the three possible pairings of the derivatives (*ẏ*, *ẏ*_2_, *ẏ*_3_) and multiply this by the third signal (not differentiated):

(7a) $$d=y_{1}({\dot{y}}_{2}-{\dot{y}}_{3})=\sqrt{3}B^{2}\Delta \dot{\phi }(t)\;{\text{cos}}^{2}\;[\Delta \phi (t)]$$

(7b) $$e=y_{2}({\dot{y}}_{3}-{\dot{y}}_{1})=\sqrt{3}B^{2}\Delta \dot{\phi }(t)\;{\text{cos}}^{2}\;[\Delta \phi (t)-120{}^{\circ}]$$

(7c) $$f=y_{3}({\dot{y}}_{1}-{\dot{y}}_{2})=\sqrt{3}B^{2}\Delta \dot{\phi }(t)\;{\text{cos}}^{2}\;[\Delta \phi (t)+120{}^{\circ}]$$

Summation of Equations (7a), (7b) and (7c), yields:

(8) $$N=d+e+f=\frac{3}{2}\sqrt{3}B^{2}\Delta \dot{\phi }(t)$$

Taking the squares of Equations (6a), (6b) and (6c), then adding them, leads to:

(9) $$D=y_{1}^{2}+y_{2}^{2}+y_{3}^{2}=B^{2}\{{\text{cos}}^{2}[\Delta \phi (t)]+{\text{cos}}^{2}[\Delta \phi (t)-120{}^{\circ}]+{\text{cos}}^{2}[\Delta \phi (t)+120{}^{\circ}]\}$$

Dividing Equation (9) into Equation (8), yields:

(10) $$Z=\frac{N}{D}=\sqrt{3}\Delta \dot{\phi }(t)$$

We can integrate Equation (10) to obtain the phase shift Δ*ϕ*(*t*) as follows:

(11) $$\Delta \phi (t)=\frac{1}{\sqrt{3}}\int \mathrm{Zdt}$$

Thus, the strain in the host structure is readily to be determined by substituting phase shift Δ*ϕ*(*t*) from Equation (11) into Equation (3).

**Figure 3.**
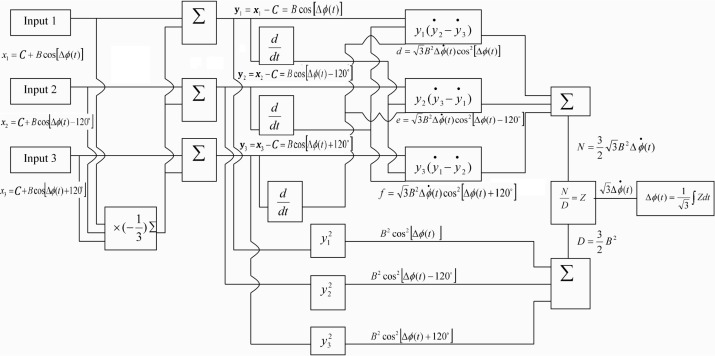
Block diagram of the phase shift demodulation.

In this study, the phase shift demodulation is performed using the commercial software Matlab. Figure 3 shows the block diagram of the demodulation process.

## 4. Numerical Example

To demonstrate the capability of the proposed methodology in demodulating the phase shift, a numerical example is presented. In the numerical example, the phase shift is assumed to be a sinusoidal function with dual angular frequencies of 34π and 50π as follows:

(12) Δϕ(t)=sin(34πt)+sin(50πt)

Substituting Equation (12) into Equations (4a), (4b) and (4c) leads to the three outputs of the 3 × 3 coupler:

(13a) $$x_{1}=C+B\text{cos}[\text{sin}(34\pi t)+\text{sin}\;(50\pi t)]$$

(13b) $$x_{2}=C+B\text{cos}[\text{sin}(34\pi t)+\text{sin}\;(50\pi t)-120{}^{\circ}]$$

(13c) $$x_{3}=C+B\text{cos}[\text{sin}(34\pi t)+\text{sin}\;(50\pi t)+120{}^{\circ}]$$

**Figure 4.**
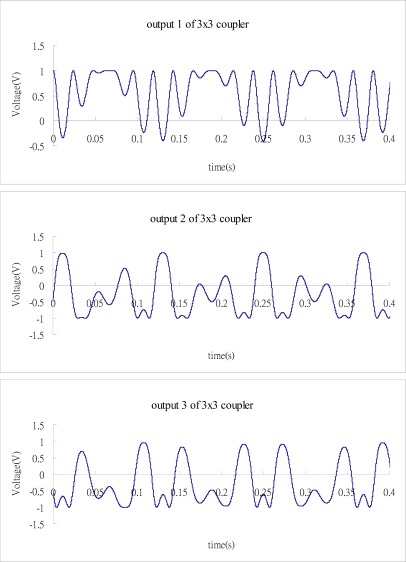
Three outputs of the 3 × 3 coupler.

Figure 4 shows the three outputs of the 3 × 3 coupler where C and B are taken to be 0 and 1, respectively.

**Figure 5.**
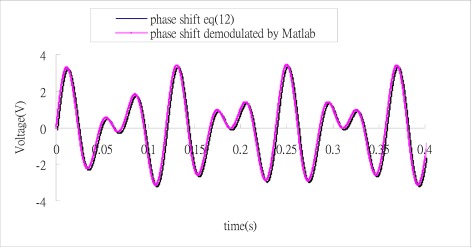
Comparison of the demodulated phase shift and exact phase shift.

Substituting the numerical data of the three outputs from Figure 4 into Matlab software, follows the process as shown in the block diagram of Figure 3 to demodulate the phase shift. Figure 5 illustrates the results of phase shift demodulated by Matlab, and compares with the exact phase shift Equation (12). It appears that the demodulated phase shift is almost the same as the exact phase shift.

## 5. Experimental Tests

**Figure 6.**
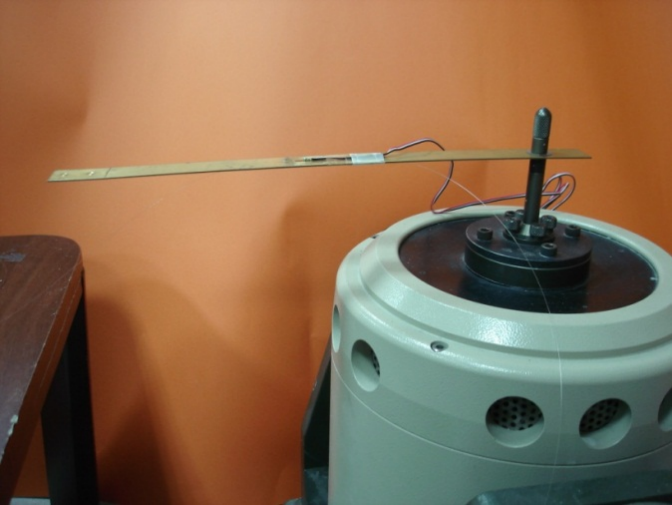
Cantilever beam mounted on a shaker.

A cantilever beam subjected to base excitation is considered in the experimental test. The beam of length L = 285 mm, width b = 20 mm, thickness h = 1 mm is made of copper with elastic modulus E = 120 GPa, density *ρ* = 8,740 kg/m^3^. An optical fiber is surface bonded to the middle of the cantilever beam as the sensing fiber of the Mach-Zehnder interferometer. The percentage of the strain in the test specimen actually transferred to the optical fiber is dependent on the bonding length [20]. The bonding length is *L_f_* = 60 mm in this work. The material properties for the optical fiber are [21]: elastic modulus *E_f_* = 72 GPa, Poisson’s ratio *v_f_* = 0.17, index of refraction n_0_ = 1.45, pockel’s constants *p*_11_ = 0.12, *p*_12_ = 0.27, radius *r_f_* = 62.5 μm. An electric resistance strain gauge is adhered to the cantilever beam near by the optical fiber. The optical fiber sensing system is a Mach-Zehnder interferometer with a 3 × 3 coupler as shown in Figure 2, operating at the wavelength of *λ* = 1,547.28 nm. The cantilever beam is mounted on a shaker as shown in Figure 6. The shaker is capable of providing maximum of four different frequencies in the same test. The natural frequency of a cantilever beam can be calculated using the following equations:

(14a) 1+cosβLcoshβL=0

(14b) $$\omega_{i}=\beta_{i}^{2}\sqrt{\frac{\mathrm{EI}}{\rho A}}$$

where *β_i_* is the root of Equation (14a); *E*, *ρ*, *L*, *A*, and *I* are the Young’s modulus, density, length, cross section area and moment of inertia of the cantilever beam, respectively.

The first five natural frequencies for the testing cantilever beam are 7.37 Hz, 46.21 Hz, 129.39 Hz, 253.63 Hz and 419.23 Hz, respectively. In the experimental test, the cantilever beam is excited by a shaker with different frequencies.

### 5.1. Test Case 1

**Figure 7.**
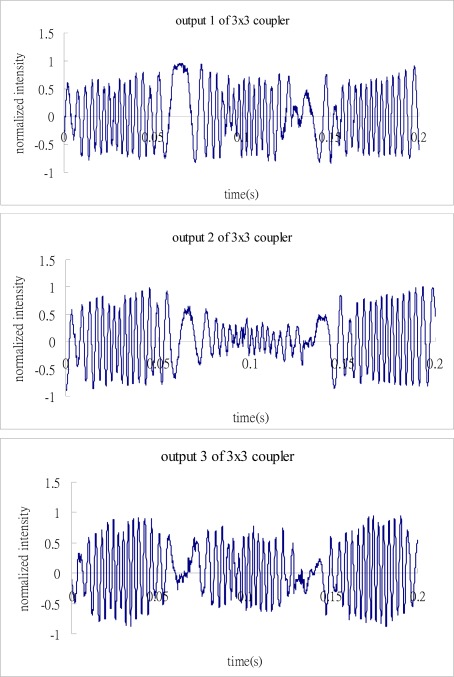
Three outputs of the 3 × 3 coupler with excitation frequency of 7 Hz.

The cantilever beam is excited by a shaker with the frequency of 7 Hz which is approximate to the first natural frequency of 7.37 Hz. Figure 7 shows the signals of the three outputs of the 3 × 3 coupler.

**Figure 8.**
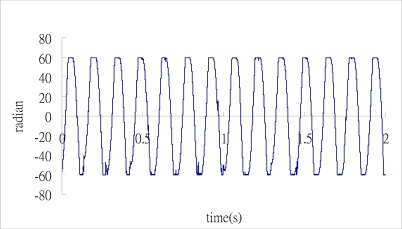
Demodulated phase shift with excitation frequency of 7 Hz.

**Figure 9.**
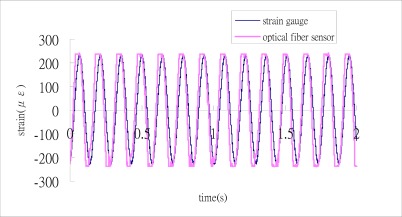
Dynamic strain of a cantilever beam subjected to base excitation frequency 7 Hz.

Substituting the three output signals of the 3 × 3 coupler as shown in Figure 7 into the Matlab software, performs the phase shift demodulation as shown in Figure 3. The result of the demodulated phase shift is presented in Figure 8. Substituting the phase shift Δ*ϕ*(*t*) from Figure 8 into Equation (3), leads to the determination of the dynamic strain of the cantilever beam. The dynamic strains obtained by the optical fiber sensor are compared with the results of the strain gauge as shown in Figure 9. Good agreement is achieved between these two sensors.

### 5.2. Test Case 2

**Figure 10.**
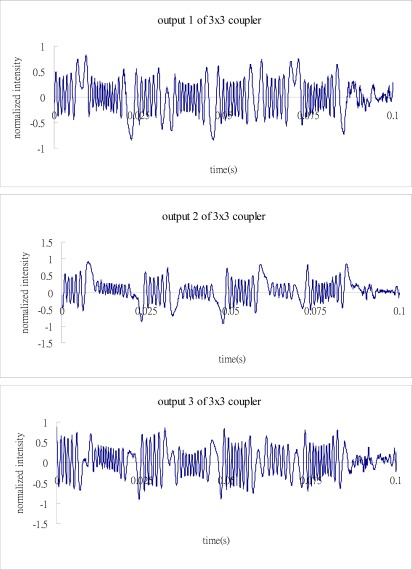
Three outputs of the 3 × 3 coupler with dual excitation frequencies of 7 Hz and 40 Hz.

The cantilever beam is excited by the shaker with dual frequencies of 7 Hz and 40 Hz, respectively. The three output signals from the 3 × 3 coupler are plotted in Figure 10.

**Figure 11.**
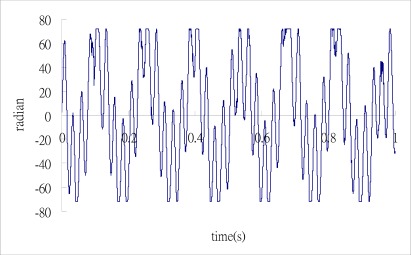
Demodulated phase shift with dual excitation frequencies of 7 Hz and 40 Hz.

**Figure 12.**
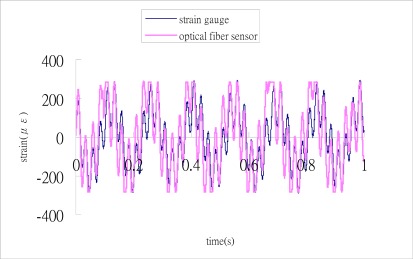
Dynamic strain of a cantilever beam subjected to dual excitation frequencies of 7 Hz and 40 Hz.

Substituting these three output signals of the 3 × 3 coupler as shown in Figure 10 into Matlab software, conducts the phase shift demodulation. The result of the phase shift is illustrated in Figure 11. Substituting the phase shift Δ*ϕ*(*t*) from Figure 11 into Equation (3), leads to the dynamic strain of the cantilever beam. The dynamic strains obtained by the optical fiber sensor are compared with the results of the strain gauge as shown in Figure 12. Reasonable agreement is observed between these two sensors. The difference of dynamics strains measured by the optical fiber sensor and strain gauge shown in Figure 12 is about 10 %. The discrepancy can be attributed to the noise of the signals.

## 6. Conclusions

Optical fiber sensors have been demonstrated for their capability to measure the dynamic responses of structures. They permit continuous monitoring of the integrity of the host structures. An optical fiber system has been developed for dynamic sensing in real time. This was done using a Mach-Zehnder interferometer incorporated with a 3 × 3 coupler for strain sensing under dynamic loading. In this work, the phase shift demodulation of the Mach-Zehnder interferometer is carried out using the commercial software Matlab. In the experimental test, the dynamic response measured by the optical fiber sensor for a cantilever beam subjected to base excitation is validated with the result of strain gauge. There is no particular restriction on the frequency of the vibrating structures in the proposed model. However, to measure the high frequency responses, it requires a data acquisition system with high sampling rate. The proposed optical fiber system is simple, inexpensive and easy to implement; moreover, it is high sensitive and accurate. These superior characteristics make it very useful and attractive for dynamic sensing.
